# Does neurokinin-1-receptor antagonist aprepitant diminish the efficacy of cyclophosphamide-based chemotherapy?

**DOI:** 10.1590/S1516-31802009000600013

**Published:** 2010-05-21

**Authors:** Carlos Eduardo Paiva, Bianca Sakamoto Ribeiro Paiva, Odair Carlito Michelin

**Affiliations:** I MD, MSc. PhD student and clinical oncologist, Oncological and Hemato-Oncological Center, Universidade Estadual Paulista (Unesp), Botucatu, São Paulo, Brazil.; II RN, MSc. PhD student and assistant nursing professor, Department of Nursing, Botucatu Medical School, Universidade Estadual Paulista (Unesp), Botucatu, São Paulo, Brazil.; III MD, PhD. Associate professor, Department of Clinics, Botucatu Medical School, Universidade Estadual Paulista (Unesp), Botucatu, São Paulo, Brazil.

To the Editor

Neurokinin-1-receptor antagonist aprepitant has been approved for clinical use based on two prospective phase III trials conducted with cisplatin-based highly emetogenic chemotherapy.^1^ It was developed for use in combination with a 5-HT3-receptor antagonist and corticosteroid in order to prevent both acute and delayed chemotherapy-induced nausea and vomiting (CINV). Warr et al.^2^ have suggested that aprepitant could be a useful antiemetic for prevention of CINV associated with moderately emetogenic chemotherapy, such as the adriamycin**/**cyclophosphamide (AC) regimen used for breast cancer treatment.

Aprepitant is metabolized primarily via cytochrome P-450 (CYP) 3A4-mediated oxidation, with minor metabolism by other cytochromes.^3^ The alkylating agent cyclophosphamide is an inactive prodrug that requires activation to form its active metabolite 4-hydroxycyclophosphamide (4-HOCP), with major participation by CYP2A6, CYP2B6 and CYP3A4.^3^ de Jonge et al.^4^ have shown that aprepitant inhibits cyclophosphamide metabolism, thus resulting in decreased formation of 4-HOCP.

It was with great interest that we read the recent paper by Yeo et al.^5^ concerning aprepitant-based CINV prophylaxis in moderately emetogenic chemotherapy. They studied a homogeneous group of Chinese breast cancer patients who underwent adjuvant AC chemotherapy (i.e. doxorubicin 60 mg/m^2^ and cyclophosphamide 600 mg/m^2^). They unexpectedly found lower incidences of neutropenia of grade ³ 3, and delay in the subsequent chemotherapy cycle. These important results led us to consider the possibility that aprepitant and cyclophosphamide drug interactions could decrease 4-HOCP formation and affect treatment efficacy.

By considering cytochrome P450 genetic polymorphisms among different breast cancer populations, we think that the clinical relevance of the aforementioned drug interaction should be urgently addressed in new studies and in different ethnic groups.
